# Addressing Alzheimer’s Risk in Racially Diverse, Low-Income Older Adults Through Telephone Health Coaching

**DOI:** 10.1093/geroni/igaa057.902

**Published:** 2020-12-16

**Authors:** Faika Zanjani, Annie Rhodes, Taylor Wilkerson, Jennifer Inker, Joann Richardson

**Affiliations:** 1 Virginia Commonwealth University, Richmond, Virginia, United States; 2 VCU, School of Social Work, Richmond, Virginia, United States; 3 VCU, Richmond, Virginia, United States

## Abstract

Preclinical Alzheimer’s disease (AD) behavioral risk reduction needs to be more fully explored at the community-level. Connecting behavior change to AD can reduce individual-level helplessness for the disease. However, behavior change targeting AD prevention factors (e.g., alcohol, depression, physical inactivity, smoking, isolation, medication management) is extremely challenging for multiple reasons, including failures in connecting AD and health behavior risk, and due to individual-level motivational, self-efficacy, and knowledge barriers. Methods: As part of the Virginia Commonwealth University iCubed Health and Wellness in Aging Population Core, 20 diverse older adults (aged 60+) living in Richmond, VA, with incomes below $12,000/year and managing either diabetes/cardiovascular symptoms, were offered weekly telephone-based health coaching for 12-weeks, providing education, motivations, self-efficacy skills, and referral services, for AD behavioral risk factors. A patient preference health coaching behavioral change strategy was implemented, where the person decides which behavioral practices to target. All study subjects completed a behavioral-psychosocial baseline and 3-month follow-up assessment. Findings: The study demonstrated feasibility for implementing health coaching within low-income racially-diverse older adults. The study sample (n=20, mean age 69 years (range: 61-77 years) was 90% African American (n=18), and 55% males (n=11). Improvement in AD knowledge (F=4.19;p=.0565); cognitive functioning (memory (F=4.19;p=.0556); delayed memory (F=2.85;p=.1086); TrailsA (F=5.60;p=.0294)), alcohol-risk (F=3.33;p=.1108) and social isolation (F=4.11;p=.0569) trends were found at 3-month follow-up. Conclusions: The findings from this study exhibit positive trends in reducing AD risk. This study creates the impetus for future large-scale investigations and dissemination of findings to improve the lives of at-risk low-income aging adults.

